# LIG-SLAM: A Lightweight Visual RGB-D SLAM for Indoor Dynamic Environments Leveraging Instance Segmentation and Geometric Information

**DOI:** 10.3390/s26102926

**Published:** 2026-05-07

**Authors:** Xingyu Chen, Jiasai Wu, Junjie Hou, Xiao Liu, Junren Sun

**Affiliations:** School of Artificial Intelligence, China University of Mining and Technology-Beijing, Beijing 100091, China; 2310402109@student.cumtb.edu.cn (X.C.); 2310402107@student.cumtb.edu.cn (J.W.); 2310402110@student.cumtb.edu.cn (J.H.); hkrgxiao@163.com (X.L.)

**Keywords:** visual-SLAM, deep learning, geometric constraints, target detection, dynamic environment

## Abstract

Traditional visual Simultaneous Localization and Mapping (SLAM) systems achieve high accuracy in static environments. However, in indoor dynamic scenes with frequent object motions, the presence of moving objects severely violates the scene rigidity assumption, often leading to significant performance degradation and tracking instability. To explicitly address this challenge, this paper introduces LIG-SLAM, a resource-efficient visual SLAM solution that extends the ORB-SLAM3 architecture. By incorporating dynamic object perception and geometric constraints, the system achieves robust localization in dynamic indoor environments, while its inference efficiency is significantly enhanced through targeted optimization. Specifically, a YOLOv5-based instance segmentation network is employed to obtain pixel-level segmentation of dynamic regions. To mitigate the erroneous rejection of static feature points, epipolar geometric constraints are incorporated to improve the accuracy of dynamic feature selection. Furthermore, a RANSAC-based depth consistency check is adopted to further enhance accuracy and alleviate the effects of epipolar degeneracy. Unlike conventional semantic SLAM frameworks, the proposed system incorporates ONNX-based optimization, thereby accelerating inference and improving real-time performance. Empirical evaluations conducted on TUM dynamic datasets indicate that the developed approach surpasses ORB-SLAM3 by a substantial margin, achieving a reduction of over 90% in terms of the Absolute Trajectory Error (ATE). Compared with existing semantic SLAM approaches, it achieves improvements in both accuracy and real-time performance, particularly in challenging indoor dynamic scenarios.

## 1. Introduction

Simultaneous Localization and Mapping (SLAM) was first introduced in the late 1980s to enable a robot to simultaneously estimate its pose and build a map of the unknown environment. Driven by the rapid evolution of autonomous vehicles, unmanned aerial systems, and mixed reality, SLAM has emerged as a cornerstone capability for modern robotics. After extensive research over the past few decades, it now provides the essential spatial awareness required for seamless autonomous navigation. Among various SLAM paradigms, visual SLAM [1] has attracted significant attention from both academia and industry in recent years due to its advantages, including low hardware cost, rich environmental perception, ease of sensor integration, and straightforward deployment. In this domain, several representative algorithms have emerged, such as MonoSLAM [2], PTAM [3], DSO [4], ORB-SLAM2 [5], and RGB-D SLAM [6].

However, conventional visual SLAM frameworks typically assume a static environment during the mapping and tracking process. By leveraging sparse feature matching, ORB-SLAM2 facilitates concurrent mapping and localization in real-time; however, its efficacy is contingent upon the environment remaining stationary. The presence of non-static entities in the scene often introduces erroneous geometric constraints, which inevitably result in accumulated tracking errors or catastrophic tracking loss. To counter these challenges, visual-inertial fusion has emerged as a robust paradigm, leveraging IMU pre-integration to bolster the system against transient visual disturbances and aggressive maneuvers. For example, ORB-SLAM3 [7] employs a tightly coupled visual inertial framework to address challenges such as rapid motion and scale ambiguity. Yet, its performance still degrades significantly in highly dynamic environments.

Recently, the advancement of deep learning has introduced new approaches to SLAM. By leveraging neural networks, deep learning methods are capable of extracting high-level semantic information from visual data. Integrating deep learning into SLAM systems enables them to perceive both geometric structures and semantic information, including object attributes and motion patterns. This provides new opportunities for robust localization in dynamic environments. For instance, DynaSLAM [8] enhances robustness by incorporating semantic segmentation and multi-view geometric constraints, effectively mitigating the influence of dynamic objects. Nevertheless, existing approaches still face limitations in real-time performance and computational efficiency.

We present a visual SLAM system designed for highly dynamic indoor environments. The system is built upon the RGB-D configuration of ORB-SLAM3 and integrates YOLOv5 [9] with multi-level geometric constraints to improve environmental representation and reduce the influence of dynamic elements.

The primary contributions of this study can be outlined as follows:

1. This work proposes a novel dynamic feature filtering strategy that tightly integrates instance segmentation with geometric constraints. By incorporating epipolar geometry, a RANSAC-based depth consistency check, and the estimated pose for joint optimization, the method improves the accuracy of dynamic feature identification while effectively preserving stable static features, even under challenging indoor dynamic conditions.
2. Unlike conventional semantic SLAM systems that rely on multi-threaded designs for semantic processing, we adopt an ONNX-based acceleration strategy to integrate semantic inference and geometric modules directly into the tracking thread. This design reduces multi-thread overhead and alleviates computational pressure on hardware resources.
3. Furthermore, this work explicitly targets resource-constrained, GPU-free platforms. The proposed system achieves efficient inference and maintains real-time performance on CPU-only devices, exploring the feasibility of semantic SLAM in low-resource environments.

The remainder of this paper is organized as follows. We first review the related work in Section 2, followed by a detailed description of the proposed method in Section 3. Experimental results and corresponding analysis are presented in Section 4, and the conclusions are drawn in Section 5.

## 2. Related Work

### 2.1. Traditional Visual SLAM

Most existing approaches address dynamic objects by removing dynamic features at the front-end of the SLAM pipeline. Still, the underlying strategies vary considerably. For example, Kim et al. [10] leverage IMU measurements to compensate for rotational motion in RGB-D cameras. Specifically, feature points from the current frame are transformed into the previous frame using the estimated rotation, and motion vectors are computed to identify dynamic features based on inconsistencies in their direction and magnitude. In contrast, Bloesch et al. [11] employ a Kalman filter to fuse visual and inertial measurements, where the IMU is used for state prediction and visual observations for state update. This enables robust tracking in dynamic and unstructured environments.

Despite these advantages, multi-sensor fusion approaches typically incur increased hardware costs and computational complexity. Furthermore, the introduction of additional sensors may lead to measurement noise and error accumulation over time.

To overcome these limitations, several approaches rely only on geometric constraints for dynamic object removal. For instance, an adaptive modeling scheme for non-static scenes was introduced in [12], where Tan et al. implemented a dynamic mechanism for the selection and refinement of keyframes in an online fashion. Their method employs a prior-guided adaptive RANSAC [13] scheme to classify features based on reprojection errors. Wang [14] proposed a geometric-centric paradigm that exploits inter-frame disparity to maintain temporal consistency. This approach facilitates the robust isolation of non-rigid entities from the static background, thereby enhancing motion segmentation in complex dynamic environments. In dynamic scenes, Scona et al. [15] developed StaticFusion, which simultaneously estimates camera motion and segments the scene into static and dynamic components by minimizing a joint photometric and geometric residual. Sun et al. [16] proposed a motion removal approach that utilizes an improved encoded representation of depth images to identify moving pixels, achieving robust performance without requiring prior knowledge of object categories. However, purely geometric methods often struggle with “slow-moving” objects that do not trigger sufficient intensity or depth inconsistencies across consecutive frames.

### 2.2. Deep Learning Visual SLAM

The rapid advancement of deep learning has significantly improved semantic understanding in robotic perception, providing new opportunities for dynamic visual SLAM. For instance, DS-SLAM [17] enhances tracking robustness by embedding a semantic-based perception module. By executing fine-grained pixel-wise classification, the system can effectively filter out dynamic outliers from the mapping process. And by combining segmentation results with motion consistency checks, it effectively filters out dynamic features. In addition, it constructs a dense semantic octree map, which is beneficial for navigation tasks. Similarly, DynaSLAM, proposed by Bescos et al., extends ORB-SLAM2 by introducing dynamic object segmentation and background inpainting, allowing the system to operate reliably in real-world dynamic scenes and achieve higher accuracy than traditional visual SLAM methods.

Beyond these approaches, RDS-SLAM [18] leverages both geometric and semantic information to improve robustness in dynamic environments. By synthesizing RGB data, depth information, and semantic masks, the system effectively filters out non-static partitions. This data-cleansing process is crucial for minimizing localization biases typically induced by mobile entities during the tracking phase. By refining local map representations, it is able to generate a more consistent and reliable reconstruction in complex scenes. Other representative segmentation-based methods, such as CFP-SLAM [19] and DRV-SLAM [20], also rely on pixel-level semantic information to suppress dynamic features and improve pose estimation accuracy. However, these methods generally depend on dense semantic segmentation, which introduces considerable computational overhead and limits their real-time applicability on resource-constrained platforms.

To address this issue, some studies adopt object detection instead of full image segmentation to reduce computational overhead. For example, Detect-SLAM [21] employs an object detection module to categorize keyframe objects into static and potentially dynamic classes. Subsequently, a motion likelihood estimation strategy is integrated with the RANSAC algorithm to detect moving objects and filter out outliers, while maintaining object-level representations in the map. Likewise, YOLO-SLAM [22] integrates the YOLOv3 detector to locate candidate dynamic objects and employs geometric constraints to further eliminate dynamic features. In particular, it uses depth consistency checks based on RANSAC to filter feature points within dynamic regions. Related methods such as OVD-SLAM [23], RLD-SLAM [24], and SG-SLAM [25] follow a similar paradigm, combining object detection with depth cues or thresholding strategies to distinguish foreground motion from static background.

In addition to detecting and removing dynamic features, recent work has explored explicitly modeling and tracking dynamic objects. For instance, Paz et al. [26] leverage conditional generative models to represent and predict the trajectories of dynamic agents, which integrates scene understanding with motion estimation. By associating and tracking moving objects across frames, these methods can exploit object motion cues to assist localization and mapping, which is particularly advantageous in highly dynamic environments.

Although effective, these approaches depend heavily on iterative optimization to address tracking and localization errors, which introduces notable computational demands and increases overall system complexity. Consequently, achieving real-time performance remains challenging for many existing SLAM systems, limiting their applicability in highly dynamic environments. Furthermore, their generalization capability in complex scenes is often limited. These challenges highlight the need for more efficient and robust approaches in both algorithm design and system implementation.

## 3. Materials and Methods

This section presents the technical details of LIG-SLAM, including the overall framework, dynamic region perception, and geometric optimization strategies. First, we describe the system architecture and processing pipeline. Next, we introduce the dynamic object detection module and the extraction of candidate dynamic regions based on instance segmentation. We then present the ONNX-based inference acceleration strategy for efficient CPU deployment. Subsequently, the epipolar geometry-based constraint is formulated to distinguish true static points from dynamic points. Finally, a dynamic point removal strategy is introduced, which integrates geometric constraints with a RANSAC-based depth consistency check to compensate for the limitations of epipolar constraints.

### 3.1. System Overview

The proposed LIG-SLAM is built upon traditional ORB-SLAM3 [7], which achieves high accuracy under the scene rigidity assumption. ORB-SLAM3 is a representative feature-based SLAM framework that operates through three parallel modules: tracking, local mapping, and loop closure. The collaboration of these components enables real-time and globally consistent estimation of both camera motion and the surrounding environment. Furthermore, ORB-SLAM3 has been extensively evaluated in public benchmark datasets, demonstrating its accuracy and robustness.

**Figure 1:**
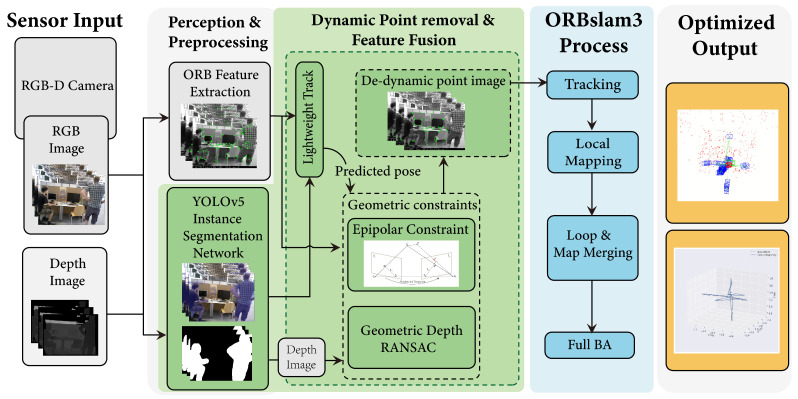
The framework of the LIG-SLAM system. The green section represents the main improvements to this system, including the addition of a detection module and a dynamic point removal module. The blue section represents the original pipeline of ORB-SLAM3, while the orange section represents the output results.

Accordingly, LIG-SLAM adopts ORB-SLAM3 as its core framework to achieve global localization and mapping. As shown in Figure 1, the system introduces two additional components: a dynamic object detection module and a dynamic feature culling module. These components are designed to filter the feature points extracted by ORB-SLAM3, enabling effective discrimination between static and dynamic features. Only static features are retained for camera pose estimation and trajectory prediction, as dynamic features can introduce significant uncertainty and degrade estimation accuracy. These modules are seamlessly integrated into the original ORB-SLAM3 framework, enhancing system functionality while preserving its architectural design and computational efficiency. In addition, epipolar geometric constraints, together with a RANSAC (Random Sample Consensus)-based depth consistency strategy, are employed to mitigate the impact of potential misclassifications in dynamic regions.

During the LIG-SLAM system operation, RGB-D frames are first converted to grayscale, followed by ORB feature extraction. In parallel (see the green region in Figure 1), a dynamic object detection module based on a YOLOv5 instance segmentation network identifies potentially dynamic objects (e.g., pedestrians). The segmentation output is represented as a binary mask, where white regions indicate candidate dynamic areas. To ensure real-time performance on CPU-only platforms, the model is deployed using the ONNX Runtime, which significantly improves inference efficiency. Recognizing that purely semantic-based filtering may lead to the misclassification of static features, a geometry and depth-based dynamic feature removal strategy is introduced. First, all feature points within candidate dynamic regions are provisionally discarded. The remaining reliable static features serve to initialize camera pose estimation and facilitate the computation of the essential matrix *E* within a lightweight tracking module. Subsequently, a RANSAC-based depth consistency check is introduced to further refine the candidate dynamic regions. Meanwhile, epipolar geometric constraints are enforced within these regions to further distinguish static features from dynamic ones. Then, as shown in the blue part of Figure 1, in this framework, both the local mapping and loop closure rely exclusively on static features for sparse map construction and loop constraint detection, maintaining consistency with the original ORB-SLAM3 system.

### 3.2. Object Detection Process

**Figure 2:**
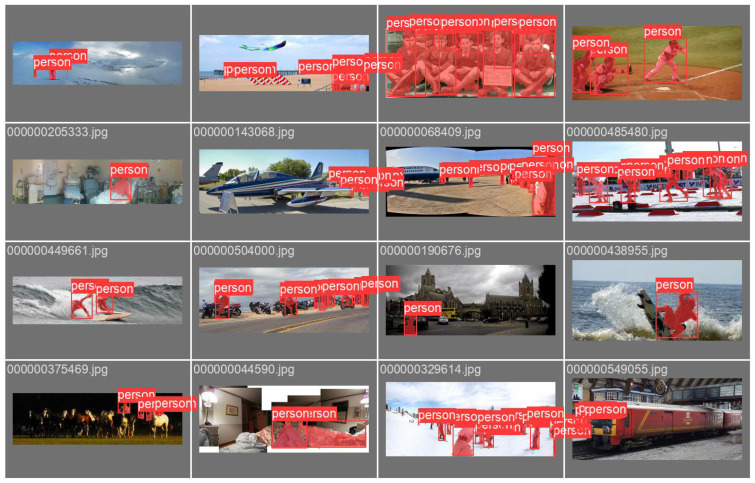
Example of instance segmentation.

In this paper, we employ the YOLOv5 [9] instance segmentation network to segment image instances. The resulting high-quality masks allow accurate delineation of individual objects while simultaneously providing semantic labels. YOLOv5-seg is selected due to its favorable trade-off between accuracy and computational efficiency, as well as its stability and wide adoption in real-world applications. In particular, it provides reliable performance under resource-constrained, CPU-only environments and supports efficient deployment through ONNX, which aligns with the design goal of this work. An example of instance segmentation is shown in Figure 2. In various scenarios, the category “person” is detected, where red bounding boxes indicate the class label and the corresponding masks roughly cover the shape of each individual.

**Figure 3:**
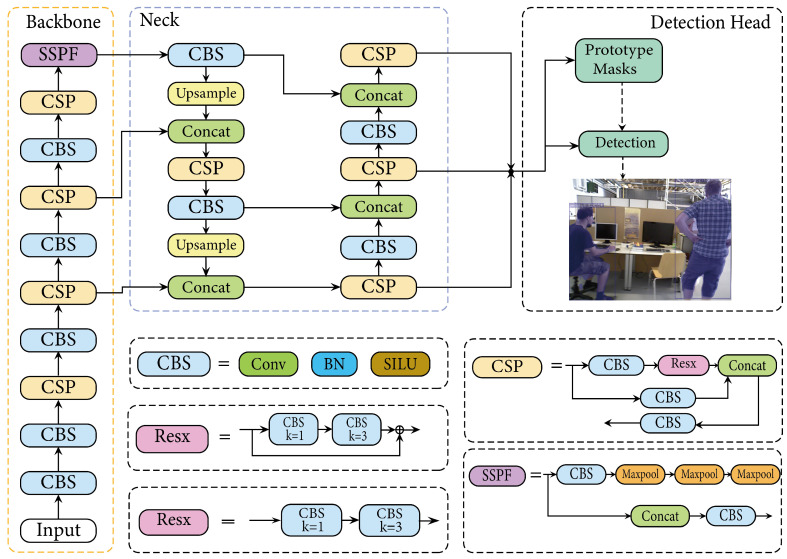
YOLOv5-seg framework.

YOLOv5-seg is developed on the basis of the single-stage detector YOLOv5, inheriting its real-time object localization and classification capabilities while introducing a prototype-based mask branch to achieve pixel-level instance segmentation. Its optimization objective comprises three decoupled components: target localization, category recognition, and semantic mask prediction. Consequently, the total loss function of YOLOv5-seg is defined as:

$$Loss_{Total}=Loss_{cls}+Loss_{box}+Loss_{obj}+Loss_{mask}$$

where $Loss_{cls}$, $Loss_{box}$, $Loss_{obj}$, and $Loss_{mask}$ represent the classification error, the bounding box regression error, the objectness estimation error and the pixel-wise segmentation error, respectively. This multi-task learning framework ensures the simultaneous optimization of detection precision and mask quality. The architecture of YOLOv5-seg is shown in Figure 3. Unlike the RoI-based FCN in Mask R-CNN [27] used by some semantic SLAMs such as DynaSLAM [8], YOLOv5-seg utilizes a Protonet branch to generate a set of *k* global prototype masks (e.g., k=32) at a resolution of m×m. Simultaneously, the detection head predicts a set of mask coefficients for each candidate box. The final instance mask is generated through a linear combination of these prototypes and coefficients, followed by a sigmoid activation and a cropping operation based on the predicted bounding box. The $Loss_{mask}$ is calculated using binary cross-entropy between the synthesized mask and the ground truth. This design avoids the heavy computational overhead of per-region sub-networks, significantly enhancing the inference speed while maintaining robust instance segmentation performance.

Through its efficient instance segmentation mechanism, YOLOv5-seg provides high-quality pixel-level masks for subsequent processing, thereby enabling reliable filtering of dynamic features. However, the native YOLOv5-seg model is computationally demanding on CPU platforms, and even lightweight variants (e.g., YOLOv5n) may struggle to meet real-time requirements in resource-constrained settings. To address this limitation while preserving the efficiency of the ORB-SLAM3-based framework, we convert the trained YOLOv5-seg model from a PyTorch-based (v2.0.0) dynamic computation graph into a static ONNX (Open Neural Network Exchange) [28] representation. This conversion enables subsequent graph-level optimizations during deployment. In particular, convolution–batch normalization (Conv–BN) fusion can be applied to incorporate normalization parameters into convolutional weights [29,30], thereby reducing redundant operations during inference. In addition, constant folding can be employed to precompute static graph components, further simplifying the computational graph and improving execution efficiency [31]. Furthermore, training-specific components such as Dropout layers are removed during export, resulting in a streamlined inference graph. The optimized ONNX model thus provides an efficient basis for accelerated inference using engines such as ONNX Runtime, enabling seamless real-time integration with SLAM systems.

**Figure 4:**
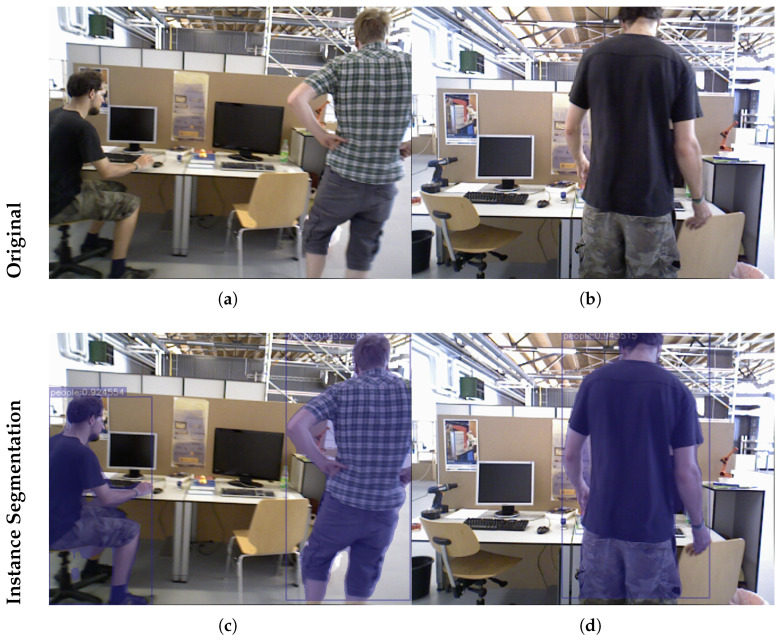
A specific example of segmentation using the YOLOv5-seg model. The (**top**) row presents the original images, while the (**bottom**) row illustrates the corresponding instance segmentation results. (**a**,**b**) represent the original images. (**c**,**d**) represent the segmented images.

The YOLOv5-seg network is trained on the COCO2017 dataset [32]. The trained model is then applied to RGB-D image sequences for instance-level segmentation. The detected dynamic objects are subsequently used to define candidate dynamic regions, facilitating downstream dynamic feature removal. The results are shown in Figure 4. In indoor environments, human motion is a primary source of dynamics. Therefore, pixels semantically labeled as “person” are likely to correspond to dynamic regions, thereby reducing the influence of dynamic objects on system accuracy.

### 3.3. Epipolar Geometry

During the object detection stage, objects that may exhibit dynamic behavior (e.g., seated individuals) are often detected. However, when relying exclusively on deep learning-based detection approaches, feature points located on human surfaces are generally treated as dynamic. Although removing all feature points within candidate dynamic regions can reduce dynamic interference, it may also result in incorrectly discarding genuine static feature points, particularly when dynamic objects cover a large area of the image. Such over-removal can degrade the accuracy and robustness of the SLAM system. Therefore, LIG-SLAM introduces epipolar geometric constraints [33] to refine dynamic feature removal. Epipolar geometry describes the spatial correspondence of a 3D point as observed from two different viewpoints. When a spatial point is imaged onto both views, the corresponding image points, the camera optical centers, and the 3D point lie on the same epipolar plane. This coplanarity condition forms the basis of the epipolar constraint that governs their geometric relationship.

**Figure 5:**
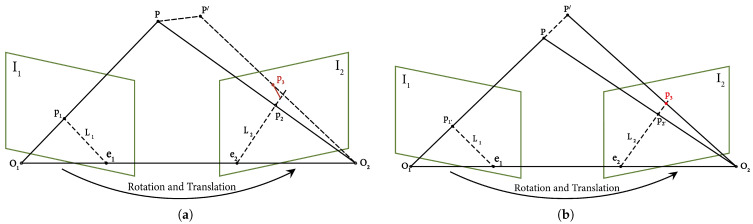
The figure illustrates the epipolar constraints associated with moving feature points. (**a**) General epipolar relationships for dynamic features. (**b**) Under certain conditions, dynamic features propagate along the epipolar line.

As shown in Figure 5, its symbol is defined as follows:

- $I_{1}$ and $I_{2}$: Image planes at two consecutive time steps;
- $O_{1}$ and $O_{2}$: Optical centers corresponding to $I_{1}$ and $I_{2}$;
- *P*: Denotes a map point observed in two consecutive frames, I1 and I2 captured by the camera from the same pose;
- $P^{\prime }$: The same point after motion (dynamic case);
- $p_{1}$: Projection of *P* onto image plane $I_{1}$;
- $p_{2}$: Projection of *P* onto image plane $I_{2}$;
- $p_{3}$: Projection of $P^{\prime }$ onto image plane $I_{2}$;
- Baseline: Refers to the straight line formed between the two optical centers, denoted as $O_{1}$ and $O_{2}$;
- Epipoles $e_{1}$ and $e_{2}$: The intersections of the baseline with image planes $I_{1}$ and $I_{2}$, respectively;
- Epipolar plane: The plane defined by $O_{1}$, $O_{2}$, and *P*;
- Epipolar lines $l_{1}$ and $l_{2}$: The intersections of the epipolar plane with image planes $I_{1}$ and $I_{2}$, respectively.

In the coordinate system of frame $I_{1}$, let K denote the camera intrinsic matrix. The relative motion between two consecutive frames is defined by a rotation matrix R and a translation vector t. For a spatial 3D point *P*, its projections onto the two image planes are denoted as pixel coordinates $p_{1}$ and $p_{2}$. Given the depths $z_{1}$ and $z_{2}$, the projection relationship can be expressed as:

(1) $$z_{1}p_{1}=KP,\;z_{2}p_{2}=K\left(RP+t\right)$$

In projective geometry, a vector is equivalent to itself multiplied by any non-zero constant. We refer to this relationship as being *equal up to a scale*, denoted by the symbol ≃. Thus, the above equations can be rewritten as:

(2) $$p_{1}≃KP,\;p_{2}≃K\left(RP+t\right)$$

Let $x_{1}$ and $x_{2}$ be the normalized coordinates of the two pixels, defined as:

(3) $$x_{1}=K^{-1}p_{1},\;x_{2}=K^{-1}p_{2}$$

Substituting Equation (3) into Equation (2), we obtain:

(4) $$x_{2}≃Rx_{1}+t$$

Left-multiplying both sides by $x_{2}^{T}t^{\wedge }$, where $t^{\wedge }$ is the skew-symmetric matrix of t, gives:

(5) $$x_{2}^{T}t^{\wedge }x_{2}≃x_{2}^{T}t^{\wedge }Rx_{1}$$

Since $t^{\wedge }t=0$, the vector $t^{\wedge }x_{2}$ is orthogonal to $x_{2}$, leading to $x_{2}^{T}t^{\wedge }x_{2}=0$. Consequently, the left side is strictly zero, and the relationship holds regardless of the scale:

(6) $$x_{2}^{T}t^{\wedge }Rx_{1}=0$$

Defining the Essential Matrix as $E=t^{\wedge }R$ and the Fundamental Matrix as $F=K^{-T}EK^{-1}$, we arrive at the epipolar constraints:

(7) $$x_{2}^{T}Ex_{1}=0,\;p_{2}^{T}Fp_{1}=0$$

Geometrically, these constraints imply that the camera centers $O_{1},O_{2}$ and the spatial point *P* are coplanar.

Based on the derived epipolar constraint, LIG-SLAM identifies dynamic features by measuring the geometric deviation. As shown in Figure 5, for a potential dynamic object, if a feature point *P* moves to $P^{\prime }$, its observed projection in frame $I_{2}$ shifts to $p_{3}$. Ideally, for a static point, $p_{3}$ should lie on the epipolar line $L_{2}$ defined by:

(8) $$L_{2}=Fp_{1}={\left[a,b,c\right]}^{T}$$

The residual distance *d* from the observed point $p_{3}$ to the theoretical epipolar line $L_{2}$ is calculated as:

(9) $$d=\frac{|p_{3}^{T}Fp_{1}|}{\sqrt{a^{2}+b^{2}}}$$

When the feature point *P* remains static, the corresponding offset distance is expected to be zero. In practice, however, it typically deviates from zero due to noise and other sources of uncertainty. Therefore, based on experience, we design an empirical threshold τ = 0.4. If *d* exceeds the predefined threshold τ, the feature point is classified as a dynamic outlier and subsequently excluded from pose estimation to ensure the stability of the SLAM system. However, as shown in the right part of Figure 5, when a feature point p moves along the viewing ray from $O_{1}$ to *P*, its projection $p_{3}$ still lies on the corresponding epipolar line $l_{2}$. In this case, the epipolar distance *d* defined in (9) becomes zero, causing the point to be incorrectly classified as static. Consequently, the discriminative capability of the epipolar constraint is significantly weakened or even fails.

### 3.4. Dynamic Object Filtering Strategy

In feature-point-based SLAM systems, the quality of extracted feature points directly influences the overall stability and performance of the system. Therefore, accurately identifying and removing dynamic features is essential. Although the dynamic regions obtained using the YOLOv5-seg network provide pixel-level accuracy superior to conventional bounding-box-based detectors, LIG-SLAM further enlarges these regions beyond the original segmentation masks. This expansion improves robustness and ensures more complete coverage of dynamic objects. However, such region inflation inevitably leads to the removal of a number of potentially valid feature points. To tackle this problem, we introduce a dynamic feature filtering strategy that integrates epipolar constraints with depth consistency checking. Specifically, we incorporate the RANSAC-based depth consistency check introduced in YOLO-SLAM [22], in conjunction with leveraging epipolar geometry to more precisely detect and remove dynamic feature points.

The overall pipeline is shown in Figure 1. Following feature extraction and semantic segmentation, feature points located in candidate dynamic regions are initially detected and temporarily filtered out. A lightweight tracking module then estimates the camera pose using the remaining high-confidence static features, from which a reliable fundamental matrix is computed. Next, a RANSAC-based depth consistency check is applied to the depth map to identify feature points whose depths fall within the depth range of dynamic objects (e.g., pedestrians). Based on the estimated pose, the system then evaluates whether a degeneracy condition occurs. If such a condition is detected, all identified dynamic feature points are removed. Otherwise, epipolar geometric constraints are incorporated to further refine the classification of dynamic and static features.

The proposed strategy is based on the following assumptions: (1) dynamic objects (e.g., pedestrians) typically occupy relatively large image regions; (2) their depth values exhibit local smoothness; and (3) within candidate dynamic regions, their depth distribution differs significantly from that of the surrounding static background.

**Figure 6:**
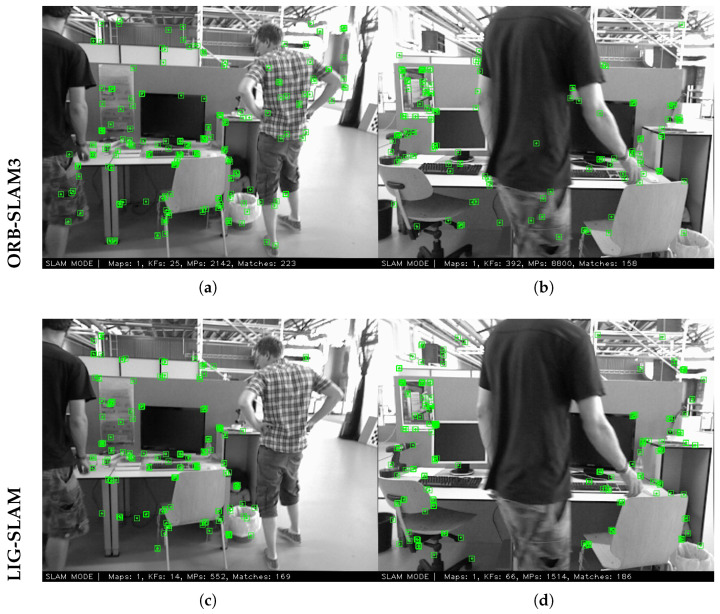
A specific example of dynamic feature point removal. In LIG-SLAM removal, feature points belonging to the dynamic object “human” are removed, while static feature points belonging to the static background surrounding the human body are preserved. (**a**,**b**) show the feature points extracted by ORB-SLAM3. (**c**,**d**) show the feature points extracted by LIG-SLAM.

Specifically, the RANSAC procedure repeatedly samples minimal subsets from the candidate dynamic feature set to estimate a depth model. Feature points whose depth values fall within an acceptable range of the dominant model are identified as belonging to dynamic objects and are consequently removed, while the remaining points are retained as static features. Finally, only the validated static features are utilized for subsequent tracking, local mapping, and loop closure detection. The complete dynamic feature removal process is summarized in Algorithm 1. The processing results are shown in Figure 6.
 **Algorithm 1:** Dynamic Feature Filtering Procedure
   **Input**: Previous frame’s feature points $P_{p}$; Current frame’s feature points $P_{c}$;
       Previous dynamic features $P_{p,dyn}$; Current dynamic features $P_{c,dyn}$; 
       Previous frame $F_{p}$; Current frame $F_{c}$; Threshold τ; Dynamic object mask *M*;
   **Output**: The set of dynamic feature points in the current frame $S_{t}$.

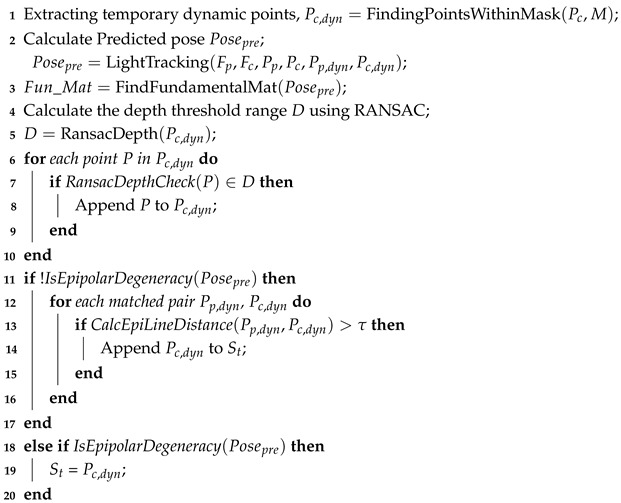


## 4. Experimental Results

This section presents the performance of LIG-SLAM on dynamic datasets. Experiments are conducted on publicly available benchmarks, including the TUM RGB-D dataset [34] and the Bonn dataset [35], which provide challenging dynamic scenarios for evaluating system robustness.

We evaluated the original ORB-SLAM3 and the proposed LIG-SLAM on a laptop equipped with an AMD Ryzen 7 8745HS CPU (3.80 GHz, 8 cores) and 16 GB of RAM. The software environment consisted of Ubuntu 20.04 running within a VMware Workstation Pro virtual machine. To test the impact of ONNX on real-time performance under resource-constrained conditions, all experiments were restricted to CPU-only settings. Meanwhile, the experiments were conducted using the YOLOv5n-seg model. The input image resolution was set to 320 × 320. The confidence threshold was set to 0.25, and Non-Maximum Suppression (NMS) was applied with an IoU threshold of 0.45 to remove redundant detections. For deployment, the model was exported to ONNX format, and inference was performed using ONNX Runtime with CPU execution provider. For the comparative analysis, only ORB-SLAM3 and the proposed LIG-SLAM were re-evaluated under the unified hardware platform, while the results of other methods (e.g., DS-SLAM, RDS-SLAM, and YOLO-SLAM) were directly adopted from their original publications. This strategy was adopted to ensure a meaningful comparison, as running these methods under CPU-only conditions would lead to severe inference delays and potentially degrade their performance. In addition, the source code of some methods is not publicly available, making fair re-implementation impractical. Considering the inherent randomness in the experimental results, each sequence in every dataset is evaluated over 10 independent runs. To mitigate the influence of outliers, the maximum and minimum values are discarded, and the final result is reported as the average of the remaining runs.

Performance is evaluated using two standard metrics: Absolute Trajectory Error (ATE) and Relative Pose Error (RPE). Specifically, ATE is evaluated using only the translational component, while RPE is decomposed into Relative Translation Error (RTE) and Relative Rotation Error (RRE) for separate analysis. To comprehensively demonstrate the performance improvements of the proposed LIG-SLAM system over the baseline ORB-SLAM3, additional statistical measures, including Root Mean Square Error (RMSE), Mean, Median, and standard deviation (SD), are reported for quantitative comparison.

### 4.1. Performance on TUM RGB-D Dataset

The TUM RGB-D dataset, developed by the Computer Vision Group at the Technical University of Munich (TUM), is a widely used RGB-D benchmark for visual SLAM research and evaluation. It encompasses a variety of indoor scenes and provides accurate ground-truth trajectories for quantitative performance assessment. For evaluation, four highly dynamic sequences are selected for primary comparison, along with one low-dynamic sequence for supplementary analysis: freiburg3_walking_xyz, freiburg3_walking_half, freiburg3_walking_static, freiburg3_walking_rpy, and freiburg3_sitting_static. Here, “freiburg3” denotes the camera setup configuration, “walking” and “sitting” indicate the dominant human activity in the scene (corresponding to high and low dynamics, respectively), and ’xyz’, ’half’, ’static’, and ’rpy’ represent different camera motion patterns. ’xyz’: camera motion along the x, y, and z directions; ’static’: the camera is kept static manually; ’halfsphere (half)’: the camera moves following the trajectory of a 1-m diameter half sphere; and ’rpy’: the camera rotates over roll, pitch and yaw axes.

The experimental results are presented in Table 1, Table 2 and Table 3. Table 1 reports the Absolute Trajectory Error (ATE), which reflects the global accuracy of the system, while Table 2 and Table 3 present the Relative Pose Error (RPE) in terms of Relative Translation Error (RTE) and Relative Rotation Error (RRE), respectively, characterizing the local accuracy.

As shown in Table 1, Table 2 and Table 3, the proposed LIG-SLAM system demonstrates clear improvements over ORB-SLAM3 in highly dynamic sequences. Specifically, as reported in Table 1, in terms of ATE, LIG-SLAM achieves an average reduction of 94.65% in RMSE and 92.63% in standard deviation (SD), suggesting improvements in both accuracy and stability under highly dynamic conditions. These gains can be attributed to the accurate localization of dynamic regions by the instance segmentation network. In addition, geometric constraints effectively remove dynamic feature interference, leading to more accurate camera pose estimation. In low-dynamic sequences, the performance gap between the two systems becomes less pronounced due to the limited motion of dynamic objects. Nevertheless, the proposed method still achieves an improvement of approximately 15%, maintaining a consistent advantage in both accuracy and robustness over ORB-SLAM3. In highly dynamic sequences, such as fr3_walking_xyz, the substantial error reduction (over 90%) reflects not merely incremental improvement but a fundamental transition from tracking divergence to robust estimation. The original ORB-SLAM3 incorporates all detected feature points—including transient features from dynamic objects—into pose estimation, leading to severe drift and potential system failure. In contrast, LIG-SLAM effectively isolates such dynamic outliers through an efficient dynamic feature filtering strategy, ensuring that trajectory estimation remains anchored to the static environment. This prevents the large error spikes observed in conventional methods and results in significant performance gains. In low-dynamic sequences, such as fr3_sitting_static, the proportion of dynamic feature points that violate the static-world assumption is relatively small. Consequently, the accuracy improvement achieved by LIG-SLAM is less pronounced compared to that in highly dynamic scenarios.

**Figure 7:**
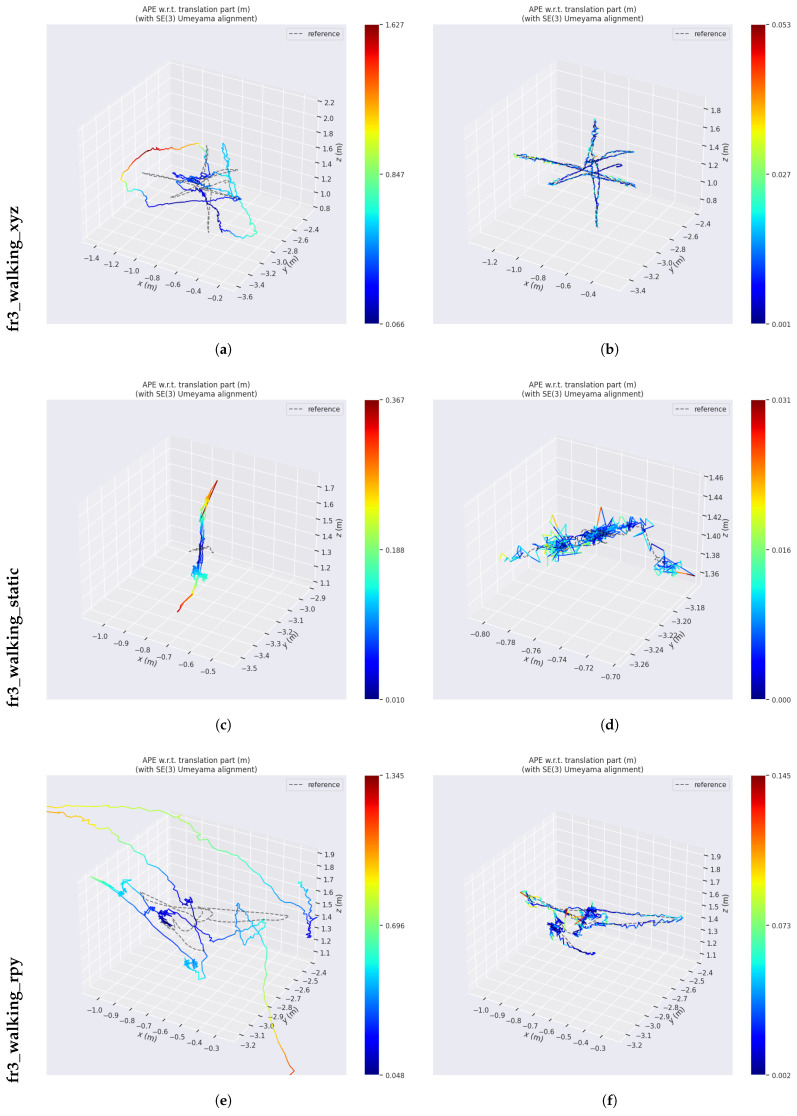

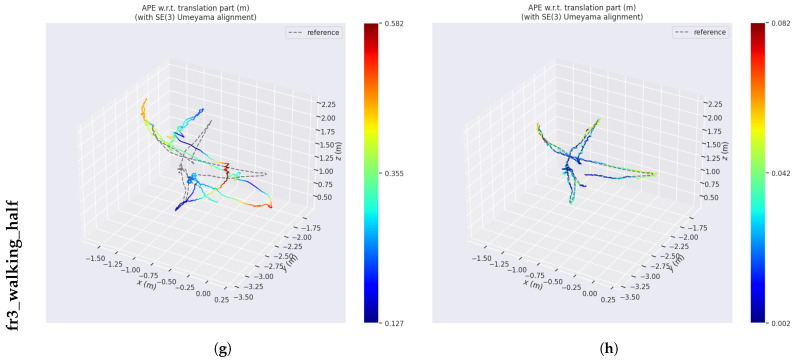
Detailed trajectory comparison between ORB-SLAM3 and LIG-SLAM (Part I). (**a**) ORB-SLAM3. (**b**) LIG-SLAM. (**c**) ORB-SLAM3. (**d**) LIG-SLAM. Detailed trajectory comparison between ORB-SLAM3 and LIG-SLAM (Part II). (**e**) ORB-SLAM3. (**f**) LIG-SLAM. (**g**) ORB-SLAM3. (**h**) LIG-SLAM.

To provide a more intuitive visualization of the pose estimation performance, a 3D trajectory comparison is presented in Figure 7. The ground-truth trajectory is shown as a gray dashed line, while the system’s estimated trajectory is displayed in color. The color variation reflects the positional error at each point. It can be observed that ORB-SLAM3 exhibits noticeable deviations in the presence of dynamic object interference, particularly in highly dynamic sequences, where the errors become significantly pronounced. In contrast, the estimated trajectory of the proposed LIG-SLAM system shows strong consistency with the ground-truth trajectory, even under highly dynamic conditions. This improvement is mainly attributed to the proposed method’s ability to effectively suppress dynamic interference, thereby enhancing both the accuracy and robustness of pose estimation.

**Figure 8:**
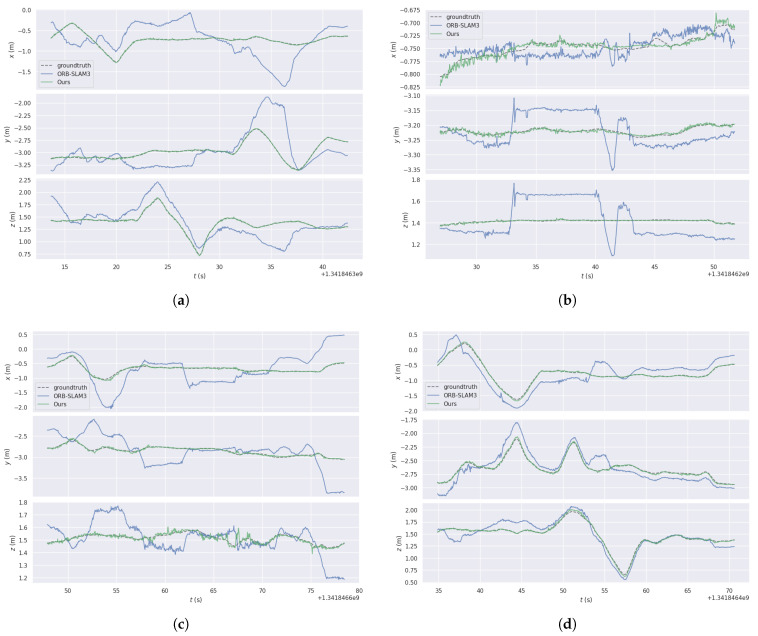
Trajectories of ORB-SLAM3 and LIG-SLAM in the xyz dimension of the TUM dataset. (**a**) fr3_walking_xyz. (**b**) fr3_walking_static. (**c**) fr3_walking_rpy. (**d**) fr3_walking_half.

As shown in Figure 8, a one-dimensional analysis is further conducted to compare the performance of the two algorithms. It can be observed that, across all sequences, ORB-SLAM3 exhibits noticeable deviations along the X, Y, and Z axes, whereas LIG-SLAM maintains higher accuracy and improved stability. These observations are consistent with the results presented in Figure 7.

To further compare the accuracy of different semantic SLAM systems, three representative approaches—DS-SLAM [17], RDS-SLAM [18], and YOLO-SLAM [22]—are selected for evaluation. The assessment is conducted using Absolute Trajectory Error (ATE) and Relative Pose Error (RPE), with Root Mean Square Error (RMSE) and standard deviation (SD) reported as the primary metrics to reflect accuracy and stability. The corresponding results are presented in Table 4 and Table 5, where bold values indicate better performance.

The results indicate that LIG-SLAM achieves competitive or superior accuracy compared with existing semantic SLAM methods, particularly in highly dynamic sequences such as fr3_walking_rpy and fr3_walking_half. In contrast, in scenarios with a higher proportion of static objects (e.g., the fr3_walking_static sequence), LIG-SLAM shows slightly lower accuracy than YOLO-SLAM; however, the performance gap remains small, and LIG-SLAM exhibits better stability. Compared with semantic SLAM systems based on bounding boxes (e.g., YOLO-SLAM), LIG-SLAM utilizes pixel-level instance segmentation to reduce redundant background regions within detection boxes, enabling more precise identification of feature points associated with dynamic objects. Moreover, instead of relying solely on geometric constraints, LIG-SLAM combines geometric information with depth analysis and pose estimation compensation from its lightweight tracking module. This multimodal strategy contributes to a more robust and stable selection of dynamic feature points, particularly in challenging dynamic environments.

### 4.2. Performance on Bonn RGB-D Dynamic Dataset

To further assess the performance of LIG-SLAM, additional experiments are conducted on the Bonn RGB-D Dynamic dataset. Released in 2019 by the Photogrammetry and Robotics Laboratory at the University of Bonn, this dataset comprises 24 dynamic sequences featuring various human activities, such as crowd movement, object transportation, and coordinated motion. Accurate ground-truth camera trajectories are provided for each sequence using the OptiTrack Prime 13 motion capture system.

**Figure 9:**
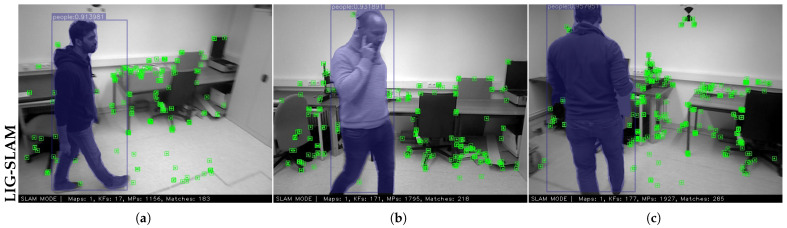
An example of dynamic feature point removal on the Bonn dataset. Dynamic objects are accurately detected, and feature points located on them are correctly filtered out. (**a**–**c**) represent three frames after instance segmentation and feature extraction.

For evaluation, five highly dynamic sequences are selected, and the results are reported in Table 6. Specifically, the ’crowd’ sequence represents a scenario in which multiple individuals move randomly within an indoor environment, while the ’person_tracking’ sequence involves the camera tracking a slowly moving person. The instance segmentation results and dynamic feature filtering performance on the Bonn RGB-D Dynamic dataset are shown in Figure 9, and the ATE comparison with ORB-SLAM3 is summarized in Table 6.

As shown in Table 6, compared with ORB-SLAM3, the proposed method delivers superior performance on most dynamic sequences, with an average ATE reduction of approximately 90%. A notable exception is the “crowd2” sequence, where the error remains relatively higher. This can be attributed to the presence of handheld objects (e.g., laptops) carried by moving individuals, whose associated dynamic feature points are not fully removed, thereby affecting the overall accuracy.

**Figure 10:**
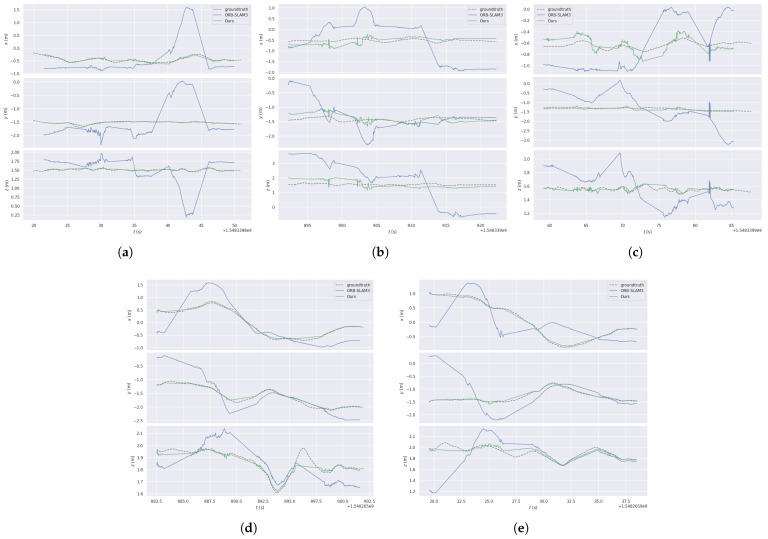
Experimental results with five frames. (**a**–**c**) Three initial stages in a crowded environment. (**d**,**e**) Subsequent processing results focusing on specific person tracking. (**a**) crowd. (**b**) crowd2. (**c**) crowd3. (**d**) person tracking. (**e**) person tracking2.

Overall, LIG-SLAM demonstrates strong performance across the majority of sequences, providing additional evidence of its effectiveness and robustness in highly dynamic environments. Moreover, the trajectory estimations along the X, Y, and Z axes, as shown in Figure 10, further highlight the superior performance of the proposed method.

### 4.3. Ablation Experiment

To evaluate the impact of different modules on system accuracy, ablation experiments were conducted to verify the effectiveness of each component. Specifically, LIG-SLAM (O) denotes the use of instance segmentation only; LIG-SLAM (O+E) denotes the combination of instance segmentation and geometric constraints; LIG-SLAM (O+D) denotes the combination of instance segmentation and depth filtering; and LIG-SLAM (A) denotes the complete system. The results are shown in Table 7. Bold values indicate better performance. It can be observed that using only the instance segmentation module (LIG-SLAM (O)) improves localization accuracy to some extent. However, this approach removes all feature points inside the detected regions without distinction, which inevitably results in the loss of static features. In the fr3_sitting_static sequence, the improvement brought by instance segmentation alone is notably limited. When combining instance segmentation with geometric constraints (LIG-SLAM (O+E)), the loss of redundant static points can be effectively mitigated, resulting in significant improvements in the fr3_walking_static and fr3_sitting_static sequences. However, under severe camera motion, geometric constraints are prone to degeneration, which reduces their effectiveness. This is clearly observed in the fr3_walking_rpy sequence, where the improvement in accuracy is less pronounced. The depth filtering module further refines the detection regions, making them better aligned with potential dynamic objects. Therefore, combining instance segmentation with depth filtering (LIG-SLAM (O+D)) can also mitigate the loss of static features to some extent. Nevertheless, since it only constrains the spatial range, its performance gains are limited in scenes with many static objects. When all three modules are jointly optimized (LIG-SLAM (A)), a substantial improvement in accuracy can be achieved, demonstrating the complementary nature of the proposed components and their ability to jointly enhance robustness and stability across different dynamic scenarios.

### 4.4. Time Analysis

In practical applications, computational efficiency is a critical performance metric for SLAM systems. To evaluate the real-time performance improvement introduced by the ONNX-based optimization in LIG-SLAM, we first compared inference speeds before and after model transformation using a consistent sequence of randomly generated images. Each backend underwent 20 warm-up iterations to eliminate latency from initial memory allocation and instruction caching. The final metrics represent the average of 100 consecutive inferences, minimizing the impact of CPU frequency scaling. It should be noted that this comparison focuses on the model inference stage only, excluding post-processing operations. As shown in Table 8, the ONNX model nearly doubles the inference speed compared to the native PyTorch version, demonstrating a significant boost in computational efficiency.

Subsequently, we evaluated the execution time of LIG-SLAM against several representative SLAM systems. To ensure the accuracy of the evaluation, we utilized the performance data reported in the original publications. The results are summarized in Table 9. Despite employing a more computationally intensive instance segmentation approach, LIG-SLAM maintains robust real-time performance without GPU acceleration, demonstrating its efficiency on CPU-only platforms. Benefiting from ONNX-based optimization, it significantly enhances frame processing speed, achieving an effective balance between real-time performance and computational overhead while maintaining modest hardware requirements.

## 5. Conclusions

This paper presents a real-time semantic SLAM framework, referred to as LIG-SLAM, designed for dynamic indoor environments. Built upon the ORB-SLAM3 architecture, the method integrates an ONNX-optimized instance segmentation module, improving dynamic object detection accuracy while preserving real-time performance. To enhance robustness, a novel dynamic feature filtering strategy is introduced, combining geometric constraints with a RANSAC-based depth consistency check to mitigate epipolar degeneracy. Experimental results on indoor datasets show that LIG-SLAM outperforms traditional methods in the evaluated scenarios in both localization accuracy and tracking stability. Ablation studies and comparisons with other semantic SLAM approaches further validate the effectiveness of the proposed deep learning and geometric modules.

Despite its strong performance, several limitations remain. In scenes with large occlusions or densely populated crowds—such as the large-area occlusions in the Bonn dataset—instance segmentation performance degrades, often leading to excessive removal of feature points and reduced accuracy. Although depth-based detection partially compensates for weak or unreliable epipolar constraints, it does not fundamentally resolve the issue, resulting in notable performance degradation in degenerate scenarios. In addition, LIG-SLAM primarily targets indoor dynamic scenes dominated by human motion, with limited exploration of more general dynamic environments. Future work will focus on extending LIG-SLAM to broader dynamic scenarios, improving robustness under extreme conditions, and exploring the construction of dense octree-based maps to meet more diverse application requirements.

## Figures and Tables

**Table 1:** Translation error (ATE, m) of ORB-SLAM3 and LIG-SLAM on the TUM RGB-D dataset.

Sequences	ORB-SLAM3				LIG-SLAM				Improvement (%)			
	RMSE	Mean	Median	S.D.	RMSE	Mean	Median	S.D.	RMSE	Mean	Median	S.D.
fr3_walking_xyz	0.8071	0.6772	0.5592	0.4391	0.0149	0.0132	0.0122	0.0070	98.15	98.05	97.82	98.40
fr3_walking_static	0.1648	0.1528	0.1612	0.0618	0.0085	0.0077	0.0071	0.0037	94.84	94.70	95.60	94.01
fr3_walking_rpy	0.5984	0.5125	0.4664	0.3088	0.0344	0.0282	0.0230	0.0196	94.25	94.50	95.07	92.56
fr3_walking_half	0.3334	0.3179	0.3091	0.1010	0.0288	0.0248	0.0217	0.0146	91.36	92.20	92.98	85.54
fr3_sitting_static	0.0106	0.0092	0.0080	0.0052	0.0078	0.0068	0.0059	0.0038	26.42	26.10	26.25	26.92

**Table 2:** Translational error (RTE, m s^−1^) of ORB-SLAM3 and LIG-SLAM on the TUM RGB-D dataset.

Sequences	ORB-SLAM3				LIG-SLAM				Improvement (%)			
	RMSE	Mean	Median	S.D.	RMSE	Mean	Median	S.D.	RMSE	Mean	Median	S.D.
fr3_walking_xyz	0.5056	0.4078	0.3456	0.3002	0.0195	0.0172	0.0161	0.0094	96.14	95.78	95.34	51.79
fr3_walking_static	0.1914	0.1287	0.0696	0.1417	0.0121	0.0111	0.0111	0.0050	93.68	91.38	84.04	96.47
fr3_walking_rpy	0.4082	0.3483	0.3653	0.3088	0.0407	0.0359	0.0328	0.0190	90.03	89.69	91.02	95.02
fr3_walking_half	0.2409	0.1697	0.0974	0.1710	0.0262	0.0237	0.0196	0.0149	89.12	86.03	79.88	91.09
fr3_sitting_static	0.0124	0.0123	0.0123	0.0158	0.0103	0.0099	0.0099	0.0030	16.93	19.51	19.51	81.01

**Table 3:** Rotational error (RRE, deg s^−1^) of ORB-SLAM3 and LIG-SLAM on the TUM RGB-D dataset.

Sequences	ORB-SLAM3				LIG-SLAM				Improvement (%)			
	RMSE	Mean	Median	S.D.	RMSE	Mean	Median	S.D.	RMSE	Mean	Median	S.D.
fr3_walking_xyz	12.074	9.7259	10.3154	7.1542	0.6472	0.5949	0.5669	0.2547	94.64	93.88	94.50	96.44
fr3_walking_static	2.9168	1.6134	0.5163	2.4272	0.4360	0.3706	0.3083	0.2289	85.05	77.03	40.29	90.57
fr3_walking_rpy	9.1939	6.5049	3.9193	6.4957	1.3191	1.1462	1.0025	0.6525	85.65	82.38	74.42	89.95
fr3_walking_half	6.005	3.7072	1.8918	4.7242	1.0975	0.9642	0.8946	0.5228	81.72	73.99	52.71	88.93
fr3_sitting_static	0.4784	0.4289	0.3999	0.2119	0.3988	0.3642	0.3695	0.1625	16.64	15.09	7.60	23.31

**Table 4:** Comparison of ATE (m) results for different SLAM systems on TUM RGB-D dataset.

Sequences	ORB-SLAM3		DS-SLAM		RDS-SLAM		YOLO-SLAM		LIG-SLAM (Ours)	
	RMSE	S.D.	RMSE	S.D.	RMSE	S.D.	RMSE	S.D.	RMSE	S.D.
fr3_walking_xyz	0.8071	0.4391	0.0247	0.0161	0.0571	0.0229	**0.0146**	0.0070	0.0149	**0.0070**
fr3_walking_static	0.1648	0.0618	0.0081	0.0036	0.0206	0.0120	**0.0073**	0.0085	0.0085	**0.0037**
fr3_walking_rpy	0.5984	0.3088	0.4442	0.2350	0.1604	0.0873	0.2164	0.1001	**0.0344**	**0.0196**
fr3_walking_half	0.3334	0.1010	0.0303	0.0159	0.0807	0.0454	0.0293	0.0148	**0.0288**	**0.0146**
fr3_sitting_static	0.0106	0.0052	**0.0065**	**0.0033**	0.0084	0.0043	0.0066	0.0033	0.0078	0.0038

**Table 5:** Comparison of RTE (m s^−1^) results for different SLAM systems on TUM RGB-D dataset.

Sequences	ORB-SLAM3		DS-SLAM		RDS-SLAM		YOLO-SLAM		LIG-SLAM (Ours)	
	RMSE	S.D.	RMSE	S.D.	RMSE	S.D.	RMSE	S.D.	RMSE	S.D.
fr3_walking_xyz	0.5056	0.3002	0.0333	0.0229	0.0426	0.0317	0.0194	0.0097	**0.0194**	**0.0094**
fr3_walking_static	0.1915	0.1417	0.0102	0.0048	0.0221	0.0149	**0.0094**	**0.0044**	0.0121	0.0050
fr3_walking_rpy	0.4082	0.3088	0.1503	0.1168	0.1320	0.1067	0.0933	0.0736	**0.0407**	**0.0359**
fr3_walking_half	0.2409	0.1710	0.0297	0.0152	0.0482	0.0362	0.0268	**0.0124**	**0.0262**	0.0149
fr3_sitting_static	0.0124	0.0158	**0.0078**	0.0038	0.0123	0.0070	0.0089	0.0044	0.0103	**0.0030**

**Table 6:** Comparison of ORB-SLAM3 and LIG-SLAM on Bonn RGB-D dynamic dataset on ATE (m).

Sequences	ORB-SLAM3				LIG-SLAM				Improvement (%)			
	RMSE	Mean	Median	S.D.	RMSE	Mean	Median	S.D.	RMSE	Mean	Median	S.D.
crowd	1.0375	0.7998	0.5144	0.6609	0.0304	0.0270	0.0240	0.0139	97.07	96.62	95.33	97.90
crowd2	1.7082	1.5353	1.4146	0.7249	0.4085	0.3683	0.3293	0.1767	76.09	76.01	76.72	75.62
crowd3	0.9999	0.9101	0.8295	0.4131	0.1304	0.1170	0.0943	0.0577	86.96	87.16	88.63	86.03
person tracking	0.6680	0.5857	0.6705	0.3211	0.0447	0.0418	0.0407	0.0157	92.86	92.86	93.92	95.11
person tracking2	0.7646	0.6281	0.5362	0.4360	0.0531	0.0507	0.0490	0.0152	93.06	91.92	90.86	96.51

**Table 7:** Comparison of ATE (m) results for ablation experiment on TUM RGB-D dataset.

Sequences	ORB-SLAM3		LIG-SLAM (O)		LIG-SLAM (O+E)		LIG-SLAM (O+D)		LIG-SLAM (A)	
	RMSE	S.D.	RMSE	S.D.	RMSE	S.D.	RMSE	S.D.	RMSE	S.D.
fr3_walking_xyz	0.8071	0.4391	0.0187	0.0111	0.0152	0.0101	0.0169	0.0093	**0.0149**	**0.0070**
fr3_walking_static	0.1648	0.0618	0.0162	0.0082	0.0092	0.0039	0.0148	0.0104	**0.0085**	**0.0037**
fr3_walking_rpy	0.5984	0.3088	0.0411	0.0273	0.0402	0.0269	0.0369	0.0228	**0.0344**	**0.0196**
fr3_walking_half	0.3334	0.1010	0.0291	0.0172	0.0289	0.0169	0.0291	0.0171	**0.0288**	**0.0146**
fr3_sitting_static	0.0106	0.0052	0.0102	0.0092	0.0079	0.0038	0.0098	0.0076	**0.0078**	**0.0038**

**Table 8:** Comparison of Model Inference Speed (CPU-only).

Model Format	Inference Time (ms)	Speedup	Hardware Platform
YOLOv5n-seg (PyTorch.pt)	24.54	1.00×	AMD Ryzen 7 8745HS
YOLOv5n-seg (ONNX)	12.52	**1.96×**	AMD Ryzen 7 8745HS

**Table 9:** Tracking Time and Hardware Platform for Different SLAM Systems.

Systems	Tracking Time per Frame (ms)	Hardware Platform
ORB-SLAM3	19.3	AMD Ryzen 7 8745H
DS-SLAM	76.5	Intel i7 CPU, P4000 GPU
RDS-SLAM	57.5	NVIDIA GeForce RTX 2080Ti
YOLO-SLAM	696.09	Intel Core i5-4288U CPU
LIG-SLAM	33.36	AMD Ryzen 7 8745H

## Data Availability

Publicly available datasets were analyzed in this study. The dataset can be found here: http://vision.in.tum.de/data/datasets/rgbd-dataset, http://www.ipb.uni-bonn.de/data/rgbd-dynamic-dataset/index.html (accessed on 4 May 2026).

## Abbreviations

The following abbreviations are used in this manuscript:

ATE	Absolute Trajectory Error
RPE	Relative Pose Error
RMSE	Root Mean Square Error
ONNX	Open Neural Network Exchange
BA	Bundle Adjustment
RTE	Relative Translation Error

## References

[1] Fuentes-Pacheco J., Ruiz-Ascencio J., Rendón-Mancha J.M. (2015). Visual simultaneous localization and mapping: A survey. Artif. Intell. Rev..

[2] Davison A.J., Reid I.D., Molton N.D., Stasse O. (2007). MonoSLAM: Real-time single camera SLAM. IEEE Trans. Pattern Anal. Mach. Intell..

[3] Klein G., Murray D. (2007). Parallel tracking and mapping for small AR workspaces. Proceedings of the 2007 6th IEEE and ACM International Symposium on Mixed and Augmented Reality, Nara, Japan, 13–16 November 2007.

[4] Engel J., Koltun V., Cremers D. (2017). Direct sparse odometry. IEEE Trans. Pattern Anal. Mach. Intell..

[5] Mur-Artal R., Tardós J.D. (2017). Orb-slam2: An open-source slam system for monocular, stereo, and rgb-d cameras. IEEE Trans. Robot..

[6] Endres F., Hess J., Sturm J., Cremers D., Burgard W. (2013). 3-D mapping with an RGB-D camera. IEEE Trans. Robot..

[7] Campos C., Elvira R., Rodríguez J.J.G., Montiel J.M., Tardós J.D. (2021). Orb-slam3: An accurate open-source library for visual, visual–inertial, and multimap slam. IEEE Trans. Robot..

[8] Bescos B., Fácil J.M., Civera J., Neira J. (2018). DynaSLAM: Tracking, mapping, and inpainting in dynamic scenes. IEEE Robot. Autom. Lett..

[9] Jocher G., Chaurasia A., Stoken A., Borovec J., Kwon Y., Michael K., Fang J., Yifu Z., Wong C., Montes D. (2022). ultralytics/yolov5: V7. 0-yolov5 sota realtime instance segmentation. Zenodo.

[10] Kim D.H., Han S.B., Kim J.H. (2015). Visual odometry algorithm using an RGB-D sensor and IMU in a highly dynamic environment. Proceedings of the Robot Intelligence Technology and Applications 3: Results from the 3rd International Conference on Robot Intelligence Technology and Applications.

[11] Bloesch M., Omari S., Hutter M., Siegwart R. (2015). Robust visual inertial odometry using a direct EKF-based approach. Proceedings of the 2015 IEEE/RSJ International Conference on Intelligent Robots and Systems (IROS), Hamburg, Germany, 28 September–2 October 2015.

[12] Tan W., Liu H., Dong Z., Zhang G., Bao H. (2013). Robust monocular SLAM in dynamic environments. Proceedings of the 2013 IEEE International Symposium on Mixed and Augmented Reality (ISMAR), Adelaide, Australia, 1–4 October 2013.

[13] Fischler M.A., Bolles R.C. (1981). Random sample consensus: A paradigm for model fitting with applications to image analysis and automated cartography. Commun. ACM.

[14] Wang R., Wan W., Wang Y., Di K. (2019). A new RGB-D SLAM method with moving object detection for dynamic indoor scenes. Remote Sens..

[15] Scona R., Jaimez M., Petillot Y.R., Fallon M., Cremers D. (2018). Staticfusion: Background reconstruction for dense rgb-d slam in dynamic environments. Proceedings of the 2018 IEEE International Conference on Robotics and Automation (ICRA), Brisbane, Australia, 21–25 May 2018.

[16] Sun Y., Liu M., Meng M.Q.H. (2017). Improving RGB-D SLAM in dynamic environments: A motion removal approach. Robot. Auton. Syst..

[17] Yu C., Liu Z., Liu X.J., Xie F., Yang Y., Wei Q., Fei Q. (2018). DS-SLAM: A semantic visual SLAM towards dynamic environments. Proceedings of the 2018 IEEE/RSJ International Conference on Intelligent Robots and Systems (IROS), Madrid, Spain, 1–5 October 2018.

[18] Liu Y., Miura J. (2021). RDS-SLAM: Real-time dynamic SLAM using semantic segmentation methods. IEEE Access.

[19] Hu X., Zhang Y., Cao Z., Ma R., Wu Y., Deng Z., Sun W. (2022). CFP-SLAM: A real-time visual SLAM based on coarse-to-fine probability in dynamic environments. Proceedings of the 2022 IEEE/RSJ International Conference on Intelligent Robots and Systems (IROS), Kyoto, Japan, 23–27 October 2022.

[20] Ji Q., Zhang Z., Chen Y., Zheng E. (2024). Drv-slam: An adaptive real-time semantic visual slam based on instance segmentation toward dynamic environments. IEEE Access.

[21] Zhong F., Wang S., Zhang Z., Wang Y. (2018). Detect-SLAM: Making object detection and SLAM mutually beneficial. Proceedings of the 2018 IEEE Winter Conference on Applications of Computer Vision (WACV), Lake Tahoe, NV, USA, 12–15 March 2018.

[22] Wu W., Guo L., Gao H., You Z., Liu Y., Chen Z. (2022). YOLO-SLAM: A semantic SLAM system towards dynamic environment with geometric constraint. Neural Comput. Appl..

[23] He J., Li M., Wang Y., Wang H. (2023). OVD-SLAM: An online visual SLAM for dynamic environments. IEEE Sens. J..

[24] Zheng Z., Lin S., Yang C. (2024). RLD-SLAM: A robust lightweight VI-SLAM for dynamic environments leveraging semantics and motion information. IEEE Trans. Ind. Electron..

[25] Cheng S., Sun C., Zhang S., Zhang D. (2022). SG-SLAM: A real-time RGB-D visual SLAM toward dynamic scenes with semantic and geometric information. IEEE Trans. Instrum. Meas..

[26] Paz D., Zhang H., Xiang H., Liang A., Christensen H.I. (2023). Conditional Generative Models for Dynamic Trajectory Generation and Urban Driving. Sensors.

[27] Lin T.Y., Dollár P., Girshick R., He K., Hariharan B., Belongie S. Feature pyramid networks for object detection. Proceedings of the IEEE Conference on Computer Vision and Pattern Recognition.

[28] ONNX: Open Neural Network Exchange. https://onnx.ai/.

[29] Ioffe S., Szegedy C. (2015). Batch normalization: Accelerating deep network training by reducing internal covariate shift. Proceedings of the International Conference on Machine Learning, Lille, France, 6–11 July 2015.

[30] Yvinec E., Dapogny A., Bailly K. (2022). To fold or not to fold: A necessary and sufficient condition on batch-normalization layers folding. arXiv.

[31] Jiang Z., Chen T., Li M. (2018). Efficient deep learning inference on edge devices. Proceedings of ACM Conference on Systems and Machine Learning (SysML’18).

[32] Lin T.Y., Maire M., Belongie S., Hays J., Perona P., Ramanan D., Dollár P., Zitnick C.L. (2014). Microsoft COCO: Common Objects in Context. Proceedings of the Computer Vision—ECCV 2014.

[33] Hartley R., Zisserman A. (2003). Multiple View Geometry in Computer Vision.

[34] Sturm J., Engelhard N., Endres F., Burgard W., Cremers D. A Benchmark for the Evaluation of RGB-D SLAM Systems. Proceedings of the International Conference on Intelligent Robot Systems (IROS).

[35] Palazzolo E., Behley J., Lottes P., Giguere P., Stachniss C. (2019). ReFusion: 3D reconstruction in dynamic environments for RGB-D cameras exploiting residuals. Proceedings of the 2019 IEEE/RSJ International Conference on Intelligent Robots and Systems (IROS), Macau, China, 3–8 November 2019.

